# NATIVITY AND DISABILITY TRAJECTORIES AMONG TWO COHORTS

**DOI:** 10.1093/geroni/igz038.1790

**Published:** 2019-11-08

**Authors:** Elizabeth Vasquez, Weihui Zhang, Anda Botoseneanu

**Affiliations:** 1 University at Albany, SUNY, Albany, New York, United States; 2 University at Albany, Rensselaer, United States; 3 University of Michigan, Dearborn, Michigan, United States

## Abstract

Nativity is an important characteristic in the context of disability in older adults, as it may influence attitudes and behaviors that can delay or accelerate the disability process. This study aims to assess trajectories of disability (defined as lower-body functional limitations, limitations in daily and instrumental activities of daily living, and gross mobility) in foreign-born and US-born Mexican Americans between 1993 and 2013. We used eight waves (1993 -2013) from the Hispanic Established Populations for the Epidemiologic Study of the Elderly (HEPESE; N=3050, mean age at baseline=73.6 (±6.8). Disability was assessed using self-reported limitations in activities of daily living (ADL /IADL). Nativity and age-at-migration were collected by self-report. We used linear and quadratic growth curve models to evaluate the trajectory of ADL/IADL disability over a period of 20 years and assessed variations by nativity status while adjusting for potential confounders. Approximately 19% of foreign-born and 17% of US-born reported having at least one ADL, while 51.5% natives and 58.7% of foreign-born had at least one IADL. Our results showed that after controlling for age at baseline, sex, marital status, self-reported health, chronic conditions, and education, foreign-born older adults were less likely to have ADL/IADL disability at baseline, but exhibited a faster rate of increase in disability over time (β =0.31 for ADL, β=0.07 for IADL). Our findings show that foreign-born older adults are accumulating disability at faster compared with their native peers, highlighting the importance of evaluating nativity differences in the health outcomes of Hispanic population at old age.

